# Vibrational study of CO, O_2_, and H_2_ Adsorbed on the CoCrFeNi (110) High Entropy Alloy Surface

**DOI:** 10.1021/acs.jpcc.4c03938

**Published:** 2024-08-19

**Authors:** Frank McKay, Andrew N. Okafor, David P. Young, Ye Xu, Phillip T. Sprunger

**Affiliations:** † Department of Physics and Astronomy, Louisiana State University, Baton Rouge, Louisiana 70803, United States; ‡ Cain Department of Chemical Engineering, Louisiana State University, Baton Rouge, Louisiana 70803, United States

## Abstract

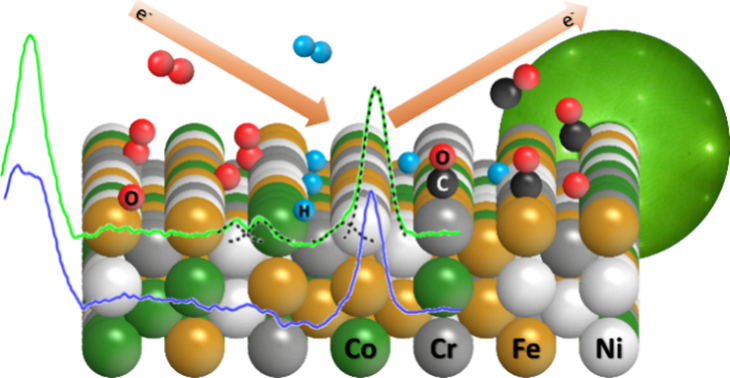

The vibrational properties of CO, O_2_, and H_2_ molecularly or dissociatively adsorbed on a CoCrFeNi(110) surface have been probed using high-resolution energy loss spectroscopy (HREELS) and modeled using density functional theory (DFT) calculations. Large (∼20 mm^3^) single-crystal, quaternary face-centered cubic CoCrFeNi was synthesized via a modified Czochralski technique. We show strong evidence that CO adsorbs primarily on bridge and on-top sites in compositionally varied local environments, which reflect the random, multielemental surface composition inherent in a high entropy alloy. A variation of adsorption sites is also found with oxygen, which exhibits two broad groups of modes. Comparison to previous photoemission and theoretical studies suggests that the higher energy modes consist primarily of local CrO_*x*_ species, while the lower energy modes are due to oxygen atoms adsorbed on other metal sites. Unlike CO and O_2_, HREELS upon H_2_ adsorption shows only two much narrower modes and is consistent with atomic adsorption on 3-fold hollow sites. The hypothesized adsorption sites for all three species are directly corroborated by our DFT calculations.

## 1 Introduction

Chemisorption on metal alloy surfaces can be complex, and yet it continues to provide a playground for understanding and developing unique catalytic behaviors. For decades, researchers have conducted fundamental investigations into the adsorption properties on single-crystal, single-element metals, and to a lesser extent, binary alloys.^1,2^ Vibrational studies of the adsorption processes on transition metal surfaces have long provided a thorough picture of the bonding and structure of adsorbates. Countless examples of studies of the adsorption of common gases can be found in the literature. Among 3d transition metals, for example, low-index surfaces of nickel have been well studied under adsorption of carbon monoxide, hydrogen,^3−7^ and oxygen.^8−10^ Other similar model systems, such as oxygen on iron^11^ and chromium,^12,13^ have been studied as well. The complexity of quaternary and higher-component high-entropy alloys (HEAs) can potentially introduce a new level of richness to catalytic selectivity and reactivity. However, fundamental studies of these more complex model systems are nearly nonexistent.^14^

This paper reports the study of surface vibrational properties of carbon monoxide (CO), coadsorption/reaction of CO + oxygen (O_*x*_), and coadsorption of CO + hydrogen (H) on a compositionally random but structurally low-index, single-crystal quaternary HEA CoCrFeNi(110) surface by high-resolution electron energy loss spectroscopy (HREELS) in comparison to density functional theory (DFT) calculations. HEAs are a relatively new class of alloys discovered by Cantor et al. and Yeh et al.^15,16^ and have been studied in both thin film^17−19^ and bulk material applications.^20−22^ Apart from their enhanced mechanical properties (i.e., hardness, strength, and ductility), HEA surfaces have been shown to have enhanced corrosion and oxidation resistance.^23−25^ Moreover, a recent study by our group has shown CoCrFeNi to be an effective electrocatalyst for the hydrogen evolution reaction (HER),^26^ exhibiting an activity that is significantly higher than its constituents and is on par with the best non-noble alternatives.^27,28^ This heightened activity is attributed to the surface oxidation behavior,^26,29^ specifically the elemental order of oxidation and the resistance to oxidation in Ni-rich sites on the surface.

To better understand the electrochemical performance, we have performed fundamental vibrational HREELS measurements on a large (110) crystalline surface of this equimolar, random HEA. This was done upon molecular/dissociative adsorption of common gas species at liquid nitrogen (LN_2_) temperatures and under ultrahigh vacuum (UHV) conditions on an atomically clean, low-index HEA surface, specifically exposure to CO, O_2_, and H_2_. In spite of the exploding research interest in employing multicomponent alloys as novel catalysts, to our knowledge, this is the first study of its kind to explore the fundamental vibrational properties of adsorbates on a HEA single-crystal surface.

## 2 Methods

### 2.1 Single Crystal Growth and (110) Orientation

The CoCrFeNi sample containing large (∼20 mm^3^) single crystal grains of the HEA was grown by a modified Czochralski technique^30^ in an RF-induction furnace. Stoichiometric amounts of the constituent elements were placed in a large alumina crucible surrounded by a tantalum sleeve that served as a flux susceptor (Figure S1 in the Supporting Information). The crucible was placed inside the RF furnace that was evacuated and backfilled with ultrahigh-purity argon gas. The elements were completely melted and mixed thoroughly. A 4 mm diameter pure single crystal tungsten rod was lowered into the top of the melt to initiate seed crystal growth. The temperature of the melt was kept just above the melting point of the HEA (*T*_m_ = 1861 °C).^31^ The rod and crucible were counter-rotated at approximately 10 rpm. A Centorr Vacuum Industries Series 3 crystal puller was used to extract the rod at a rate of approximately 3 mm/h. Afterward, a section was sliced perpendicular from the single-crystal tungsten seed rod by electrical-discharge machining (EDM). This EDM-cut ingot was then sanded and polished with a compound of alumina powder (down to 0.2 μm, 99.99%, Alfa Aesar) to preferentially reveal (<2°) a (110) surface.

### 2.2 Surface Science Experimental Methods

HREELS experiments were performed in an UHV chamber (10^–10^ Torr base pressure) equipped with a LK ELS5000-MCA HREELS spectrometer, low energy electron diffraction (LEED) optics (Specs ErLEED 1000-A), and neon ion sputtering gun. An ∼8 × 8 × 2 mm^3^ HEA disk exhibiting a (110) surface was spot-welded to a sample holder, which was suspended by 0.25 mm W wires that provided joule heating. Sample cooling was provided via an external LN_2_ reservoir. A type K thermocouple was attached to the back of the sample to monitor temperature. Prior to dosing the sample, the sample was cleaned by repeated cycles of neon ion sputtering (45 min) at room temperature and annealing (∼800 °C for 5 min). The clean sample was then cooled to −190 °C prior to dosing or taking measurements. Gas dosing was achieved via a manually controlled leak valve which was typically performed at a chamber pressure of 10^–6^ Torr for a preset time (1 L = 10^–6^ Torr·s) using only high purity gases (99.99%). Prior to LEED or HREELS measurements, the sample was flash annealed to approximately 800 °C just prior to gas exposure. HREELS measurements were conducted with an electron beam energy of 7.43 eV and a resolution of approximately 8 meV.

To increase signal-to-noise, all HREELS spectra were taken over a period of approximately 1 h in specular geometry (θ_inc_ = θ_scat_ = 55°). Due to an inherent small atomic-scale roughness, the reflectivity of our crystal was less than, for example, Ni single crystal surfaces. The resultant data, which were dominated by the dipole-active modes perpendicular to the surface, were then normalized to the elastic peak intensity, and a background was subtracted using a decaying exponential fit to data of a clean sample. All peak positions and widths reported were determined by fitting a standard Gaussian peak using the method of least-squares fitting.

To reveal bulk structure, 2D-XRD (Bruker D8 GADDS in sample spinning mode) with Cu Kα (λ = 1.5406 Å) was employed. Probing the nearer surface structure (10–15 nm), electron-backscattered diffraction (EBSD) and energy-dispersive X-ray spectroscopy (EDS) measurements (at 20 keV) were performed using a ThermoScientific Helios G5 XVe PFIB at the Shared Instrument Facility at Louisiana State University. Finally, low-energy electron diffraction (<400 eV) was utilized to elucidate surface (≲1 nm) in UHV using a reverse-view LEED optics.

### 2.3 Computational Methods

DFT calculations were performed in the generalized gradient approximation (GGA-PBE)^32^ using the Vienna Ab Initio Simulation Package (VASP).^33^ The Kohn–Sham one-electron valence states [Cr(3p3d4s), Fe(3d4s), Co(3d4s), Ni(3d4s), O(2s2p), C(2s2p), and H(1s)] were expanded in a plane-wave basis set up to 650 eV. The potentials due to the core electrons were described using the projector augmented wave method.^34^ All DFT calculations were done without spin polarization as an approximation of the ground state of the HEA, which is paramagnetic, because of the prohibitive costs of noncollinear calculations and exhaustive enumeration of possible magnitudes and directions of magnetic moments that would be needed to represent paramagnetic states.

**Figure 1:**
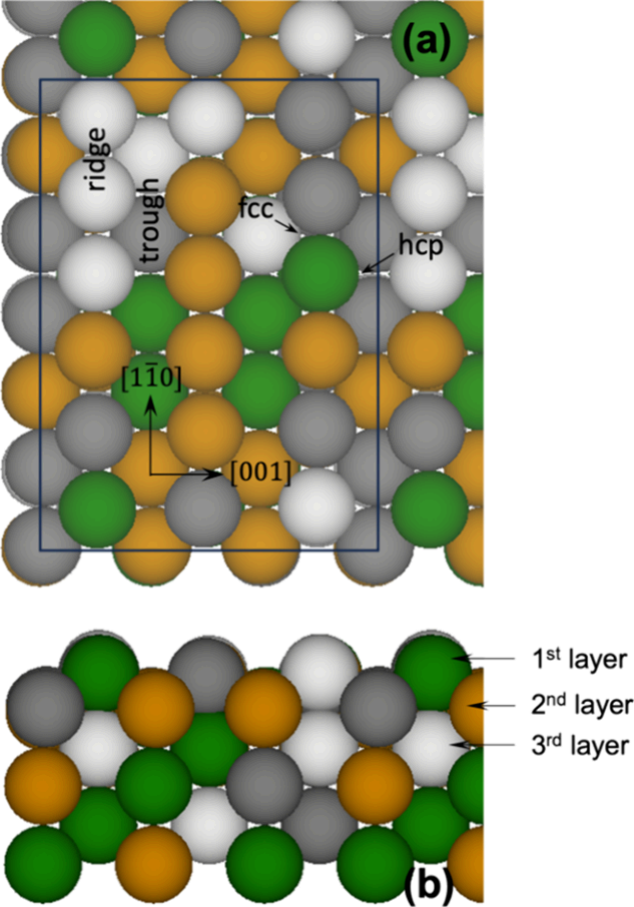
Clean (6 × 6) surface model for the (110) facet of the CoCrFeNi HEA: (a) top view; (b) side view. Color code: Co = green; Cr = dark gray; Fe = yellow; Ni = white. The surface unit cell is outlined in panel (a), with surface features and sites on the surface labeled.

The bulk HEA model was taken from our previous work, and detailed information on how it was constructed can be found therein.^26^ Briefly, a large 108-atom Co_27_Cr_27_Fe_27_Ni_27_ bulk supercell representation of the HEA was generated using the Super-Cell Random Approximates (SCRAPs) method by Johnson and co-workers.^36^ As we had done previously, we used finite surface models to capture the effects of surface heterogeneity and high adsorbate coverage on the vibrations of the adsorbates with the accuracy afforded by DFT. Just as no supercell of any practical size can fully represent the bulk HEA, no finite surface model can uniquely represent the HEA surface. As a compromise between model size and computational efficiency, we constructed a (6 × 6) surface unit cell to represent the unreconstructed (1 × 1) (110) facet of the HEA, which was cut from the bulk supercell (Figure 1). The facet consisted of a series of ridges and troughs. The slab was six metal layers thick and was separated from periodic images in the z direction by 10 Å of vacuum. The surface Brillouin zone was sampled on a 2 × 2 × 1 Gamma-centered k-point mesh. A first-order Methfessel-Paxton scheme was used to smear the electronic states with a width of 0.2 eV.^37^ Adsorbates were placed on the top of the slab only. All adsorbates and the top three metal layers in the slab were fully relaxed, while the remaining three layers were held fixed at their bulk positions. Geometry optimization was taken to be converged when the residual force in each relaxed degree of freedom in the system was lower than 0.03 eV/Å.

Calculations of the vibrational normal modes of the adsorbates and their frequencies were performed within the Atomic Simulation Environment^38^ in the harmonic approximation, using a finite difference approximation of the dynamical matrix with a two-sided displacement of ±0.01 Å. The vibrational intensities of the normal modes were calculated using a finite difference approximation of the gradient of the dipole moment perpendicular to the surface with a displacement of ±0.01 Å. A nonzero dipole gradient in the specular direction contributes intensity to a vibrational normal mode in the HEELS. A Gaussian broadening width of 60 cm^–1^ (7.4 meV) was then applied to the resulting IR spectra. The value was based on the average peak resolution obtained in our HEELS spectra.

## 3 Results and Discussion

### 3.1 Single Crystal Growth and Characterization

In previous studies, we have characterized a similar CoCrFeNi sample.^26,29^ The previous sample was found to have a face-centered cubic (*fcc*) crystal structure, with a lattice constant of 3.56 Å, consistent with the literature.^39,40^ In our previous study EBSD measurements showed smaller grains around 10–100 μm in areal size. However, for this single-crystal sample EBSD measurements (Figure S2a in the Supporting Information) show a large single grain at least 2 × 3 mm^2^ in area. In addition, EDS confirmed that in the near-surface region (<80 nm) both the small grain and single crystal sample remained compositionally nearly equimolar (±0.5%), consistent with other HEAs.^15,16^ Lastly our 2D XRD measurements (See Figure S2b in the Supporting Information) show a single-phase *fcc* structure with a lattice constant of 3.56 Å, consistent with our previous studies.

In our previous work,^26,29^ the surface of a CoCrFeNi sample was characterized by X-ray and UV photoelectron spectroscopy (XPS and UPS, respectively). Angle-dependent XPS confirmed the small-grain sample was equimolar in the surface selvage (<2 nm), revealing no preferential concentration of surface elements. The focus of our previous publications was the characterization of this alloy under oxidation and how that related to its electrochemical activity. In those studies, theoretical calculations and XPS confirmed the propensity of oxygen to bind to the surface in the order Cr > Co ≈ Fe > Ni, forming oxides in all but Ni with increasing exposure.

**Figure 2:**
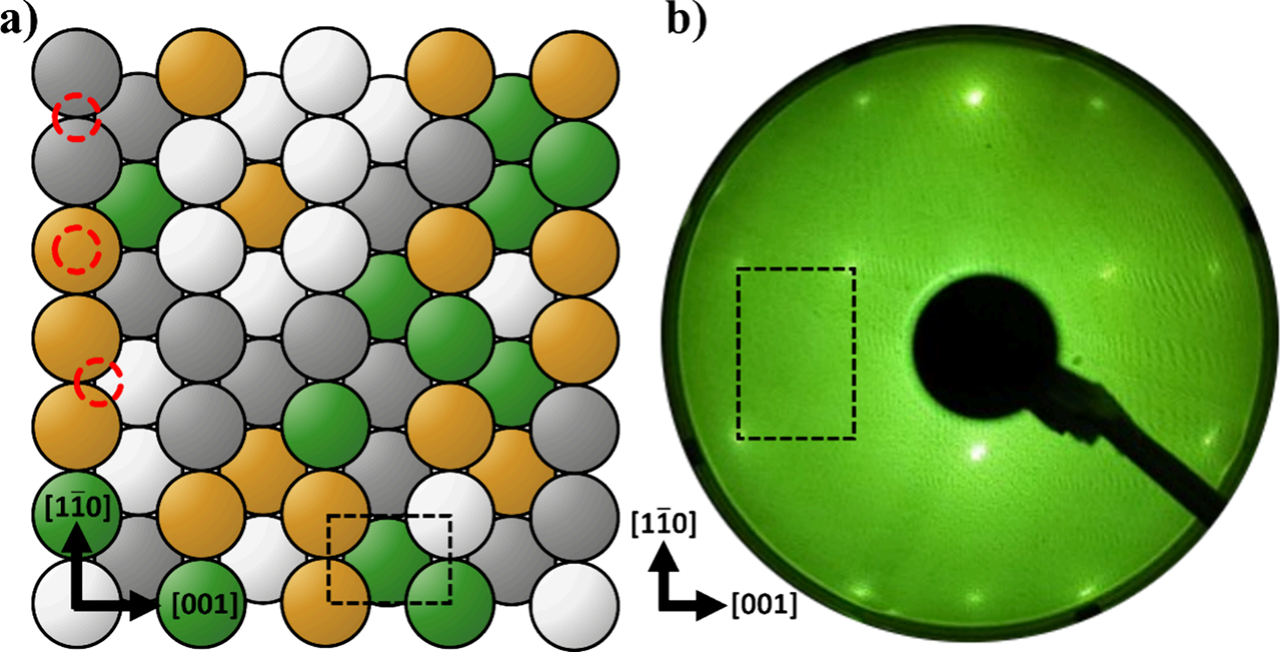
(a) Schematic of the fcc structure of a random (110) quaternary HEA alloy, where different colors indicate different elements. Possible adsorption sites are indicated by dashed-red circles in (top to bottom) bridge, on-top, and 3-fold hollow configurations. (b) LEED pattern of a clean CoCrFeNi(110) surface at LN_2_ temperatures, *E*_inc_ = 221 V. The dashed rectangle represents a multiple of the unit cell.

Based on these studies, the alloy surface contains a random distribution of elements as illustrated in Figure 2a. While there exists structural symmetry in this alloy, there exists no compositional short or long-range order. As seen from the model, each possible adsorption site on the surface is different in the elemental composition of the local environment.

### 3.2 LEED

The LEED pattern of a single-crystal, clean CoCrFeNi(110) surface is shown in Figure 2b. This pattern shows the typical [110] diffraction spots that indicate a large single crystal grain of a *fcc* structure nearly perpendicular to the surface. The size of the crystal grain was confirmed by translating across the crystal (∼4 mm) without a change in the LEED pattern. As seen in Figure 2b, the width of the diffraction spots at −190 °C was not as sharp as single element crystal surfaces, due to apparent roughness of the surface, or small local relaxations. A certain amount of in- and out-of-plane surface roughness is expected due to the difference in atomic composition of the surface which causes small local distortions in the lattice.^41^ Upon exposure to O_2_, the LEED diffuse background increases (i.e., short-range symmetry attenuates) until nearly no apparent (110) symmetry can be discerned. Additionally, no additional superstructures were seen upon O_2_ exposure (Figure S3a–c in the Supporting Information), consistent with the compositional randomness of the surface and preferential oxidation. The LEED patterns (Figure S3d–f in the Supporting Information) upon H_2_ dosage (H-adsorption) were indiscernible from the pattern of a clean sample. No noticeable change in spot intensity or additional superstructures could be seen upon H_2_ dosage, even up to 100 L H_2_.

### 3.3 CO Exposure

**Figure 3:**
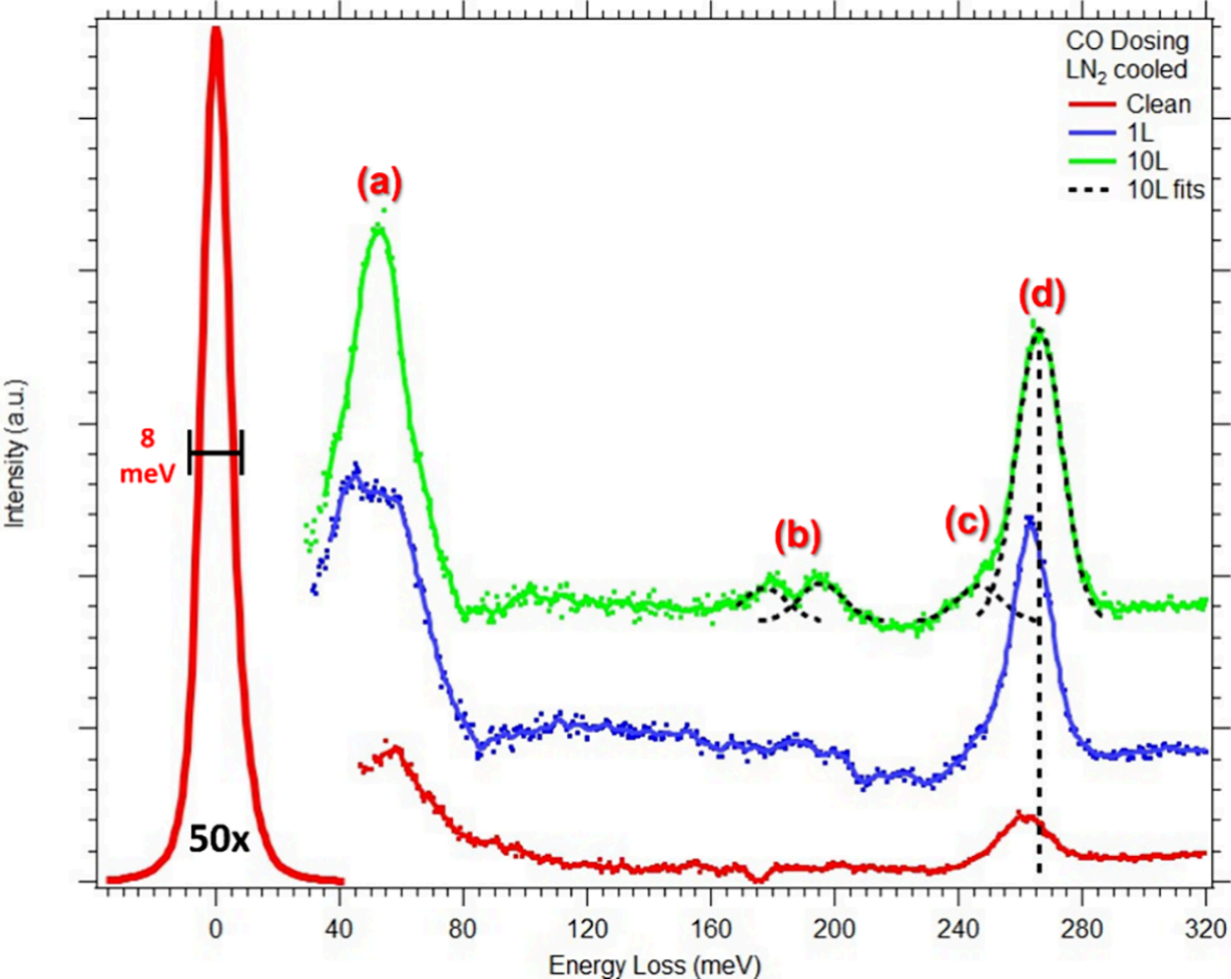
HREELS difference spectra of CoCrFeNi(110) sample: clean (red), 1 L CO (blue), and 10 L CO (green). Fits for 10 L CO are displayed in black. Vertical dashed line indicates peak position at 10 L dosing. Four distinct modes are labeled here corresponding to (a) M-CO mode, (b) to C–O 3-fold hollow sites, (c) to C–O bridge stretching modes, and (d) to C–O on-top stretching modes.

HREELS difference spectra of the CoCrFeNi(110) sample when dosed with CO can be seen in Figure 3. Two strong vibrational modes can be seen at all exposures, one around 50 meV and another around 260 meV. These energies are characteristic of the metal–carbon monoxide (M-CO) modes (a) and the carbon–oxygen (C–O) stretching modes (d), respectively, which is indicative of molecularly adsorbed CO at on-top positions.^7,42,43^ At 10 L dosing, considered as saturation coverage, two additional modes can be seen around 180–190 meV (b), which correspond to adsorption on 3-fold hollow sites.^44^

Four distinct modes can be seen in Figure 3 on the displayed fits labeled a-d at 10 L CO. In the dominant C–O stretching modes (d), a shoulder at lower energy can be seen at higher coverages. As typical with CO adsorption on other transition metal surfaces, this is indicative of additional CO adsorbed on bridge sites (c), as indicated around 245 meV. However, instead of seeing two distinct modes, as may be expected on a pure metal surface, these features overlap here because they are broad, having a full-width a half-maximum (fwhm) of about 17 meV, twice that of the elastic peak at 8 meV. Upon increased CO exposure, the energy of the C–O stretching modes (c and d) gradually increases in position, presumably due to repulsive adsorbate–adsorbate interactions. This perspective can be seen with the dashed vertical line in Figure 3, which is located at the position of the main C–O stretch mode (d) at 10 L CO dosing. The location of the M-CO loss feature (a) did not change significantly but shows a similar broadness to the C–O stretch peaks. As discussed later, similar results can be seen in our DFT calculations.

This increase in the C–O stretch peak positions with exposure is typical for CO adsorption on metal surfaces bonded to on-top or bridge sites.^7,45,46^ Since each of the four elements in this alloy has a locally different *d*-orbital filling (see Figure S4 in the Supporting Information), the subsequent difference in back-bonding would slightly change the energy of the ensuing vibrational modes depending on the local elemental bonding site/element. That is, the exact energy of the vibrational mode depends not only on the type of adsorption site, as in single-element systems, but reflects subtle changes of the local electronic environment. In our sample, which is an alloy with a compositionally disordered surface and a large variety of different local environments, the difference in local environments for adsorption can notably affect the individual vibrational energy of compositionally distinct adsorption sites, causing a broadening of the observed vibrational energy for each mode at saturation that exceeds the width of the elastic peak. The large widths of these peaks reflect a manifold of slightly differing vibrational energies due to bonding in slightly differing electronic environments.

### 3.4 Oxygen Exposure

**Figure 4:**
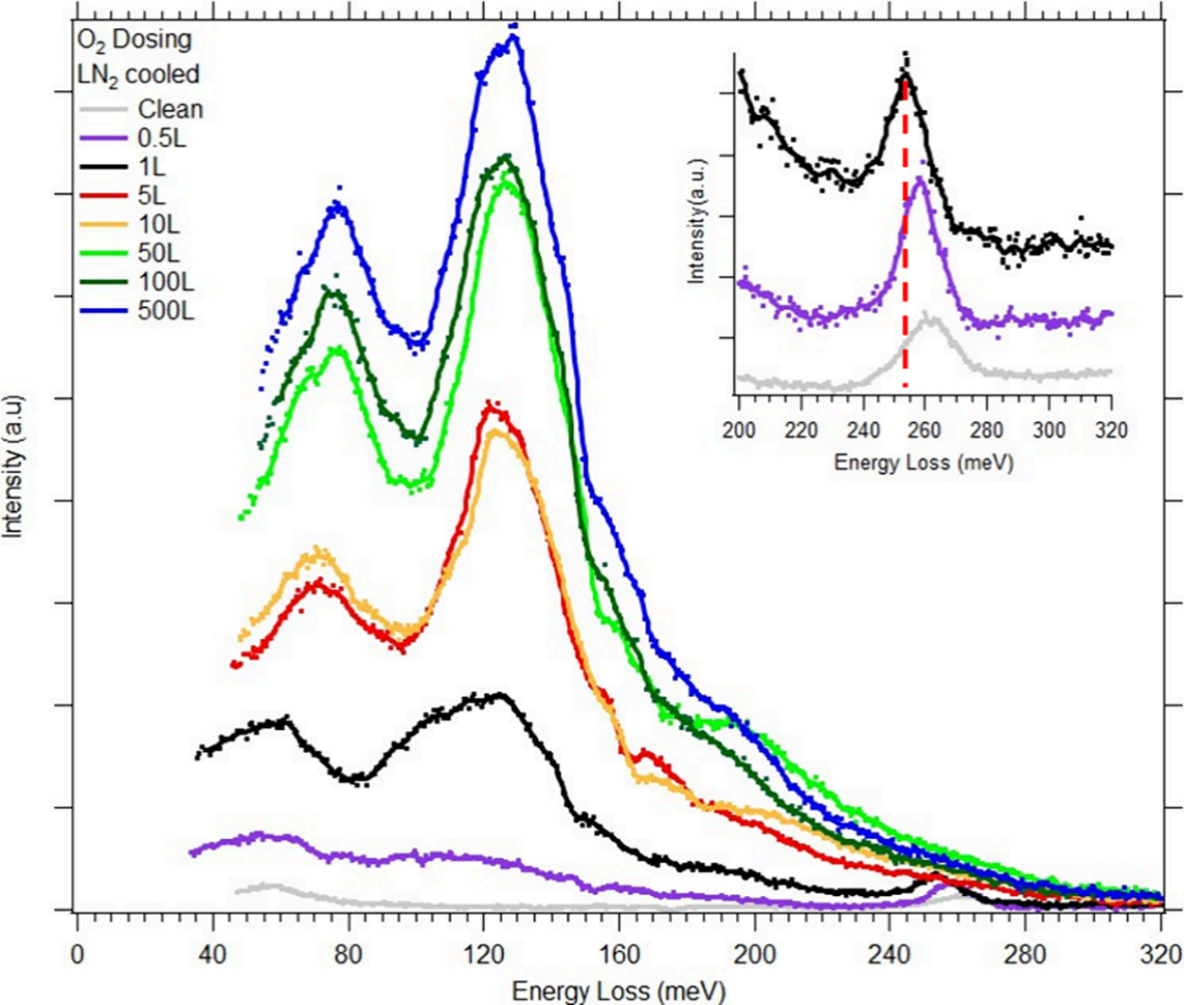
HREELS spectra of CoCrFeNi(110) sample upon dosing up to 500 L O_2_. Inset displays C–O stretching modes at low oxygen dosing (Clean, 0.5 L, and 1 L O_2_). Clean indicates spectra prior to dosing. Dashed red line indicates shift in peak position at additional O_2_ dosing.

HREELS spectra of the CoCrFeNi(110) sample upon dosing of O_2_ are presented in Figure 4. Upon increasing exposure to oxygen, two strong features develop around 72 and 125 meV, which we assign to different metal–oxygen/oxide (M-O) and metal-oxide species (M-O_*x*_) modes, respectively.^11,12^ These peaks increase in intensity as oxygen surface concentration is increased. This development of oxides is consistent with our previous studies,^26,29^ wherein it was determined that oxygen passivates the surface, beginning on Cr first, quickly followed by Fe and Co, but not on Ni.

In our previous XPS studies^26^ we showed evidence of oxide species formation in the case of CrO_*x*_ and FeO_*x*_. In addition, by calculation of the differential reduction free energy (*dG*^red^) of adsorbed oxygen, the propensity of oxygen to bond to sites rich in Cr and Fe was determined to be higher than to sites rich in Co or Ni.^26,29^ This same general trend can be seen in Figure 4 due to the appearance of additional (M-O_*x*_) oxide modes at low dosing. Of the four elements present in this HEA system, normally only Co, Cr, and Fe can develop higher oxide modes (M-O_*x*_ where *x* > 1).

Due to traces of residual CO in the UHV chamber, most HREELS spectra show evidence of small amounts of CO adsorption, even on a cleaned sample, appearing after being flash annealed. This was due to the duration of the scans. The surface was found to take up CO so strongly that within 15 min of flashing, CO modes, which have a large dipole moment, appeared in the spectra even at low UHV pressures. Since scans needed to be over an hour to achieve sufficient signal-to-noise, most scans showed evidence of some CO adsorption, unless otherwise indicated.

**Figure 5:**
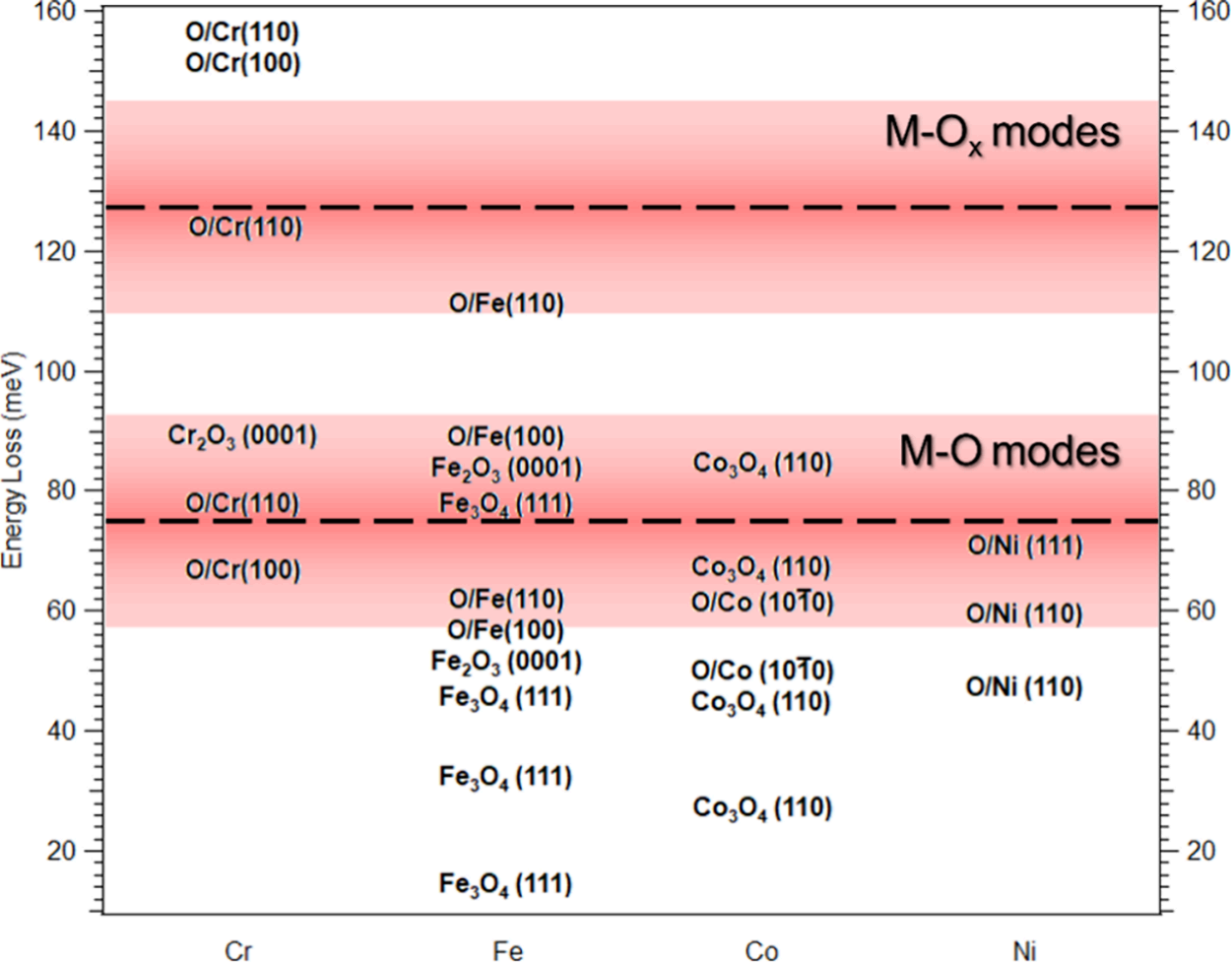
Literature values for vibrational modes of adsorption of oxygen on single element systems of Cr, Fe, Co, and Ni and Fuchs-Kliewer phonon modes from refs (8), (11−13), and (39−46). Red shaded regions represent peak positions with fwhm from our Figure 4 CoCrFeNi(110) EELS measurements.

As with intentional CO dosing, the width of the peaks is indicative of the variety of different local environments for adsorption on this surface, reflecting the combinational myriad of elemental atoms. The fwhm of these features averages around 35 meV for both M-O and M-O_*x*_ modes, which was four times the width of the elastic peak at 8 meV. To illustrate the effect of the local environment on vibrational modes, a comparison to monometallic systems is warranted. In Figure 5, literature values for observed vibrational modes of oxygen on single-element systems of Co, Cr, Fe, and Ni and optical phonon modes of Co_3_O_4_, Cr_2_O_3_, and Fe_2_O_3_/Fe_3_O_4_ are compared to our measurements.^8,11−13,47−54^ The shaded regions in Figure 5 represent the fwhm of the observed M-O and M-O_*x*_ modes for 5 L O_2_ and higher, where the center of the red region is the average position.

As mentioned before (see Figure 2a), this sample has a random distribution of elements within the surface selvage. Some adsorption sites may consist of just one element, while the majority are a combination of multiple elements. Figure 5 shows just some of the different vibrational modes that could exist on our surface. All these systems from literature have some modes that overlap with our measured peaks. In particular, modes from oxygen on Cr single-element systems have a much higher vibrational energy than the other single-element systems in this comparison. Based on this and our previous work,^26^ which showed that Cr is the first to be oxidized, we conclude that the M-O_*x*_ modes are primarily composed of adsorption sites that are rich in Cr. As discussed later, our experimentally measured vibrational modes can be compared to those obtained by our DFT calculations in Figures 7d and 8b.

As previously discussed, even spectra for a “clean” surface (prior to dosing) show evidence of the adsorption of residual CO by two vibrational modes present at 56 and 262 meV, which correspond to the M-CO and C–O stretch modes respectively, as seen in the inset of Figure 4. However, the CO modes vanish above 1 L dosing of O_2_. Additionally, as indicated by the vertical dotted line, the C–O mode peak position redshifts after dosing of oxygen from 261.5 meV (clean) to 253.4 meV (1 L O_2_). This decrease in energy is consistent with other studies that have shown a change in the energy of the C–O stretch mode upon adsorption of other gases,^46,55^ due to adsorbate–adsorbate interactions. As with CO dosing, the width of the C–O peak is around 15 meV, indicating a variety of different local environments of adsorption.

At low oxygen dosing, this behavior can be compared to other systems. In a previous STM study,^56^ it was found that when CO and O_2_ were simultaneously dosed onto a Ni(110) surface, they competitively adsorbed, and phase-segregated into oxygen-rich and CO-rich islands. However, in the present CoCrFeNi(110) system, LEED shows no evidence (no additional superstructures) that O and CO phase-segregate at low oxygen coverage (Figure S3 in the Supporting Information). On spectra of 5 L and higher, no evidence of any CO adsorption is present. This can be seen by the absence of a feature centered around 56 meV and the disappearance of the C–O stretch mode at 262 meV. This indicates competitive adsorption between oxygen and CO at high dosing and possible CO oxidation,^57^ forming CO_2_ and/or subsequent desorption.

### 3.5 Hydrogen Exposure

**Figure 6:**
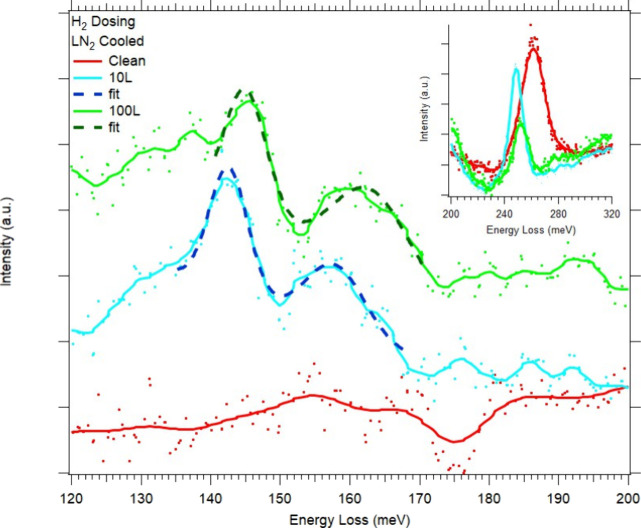
HREELS spectra of CoCrFeNi(110) sample upon dosing up to 100 L H_2_. Inset shows effect of H_2_ dosing on C–O vibrational mode.

HREELS spectra of the CoCrFeNi(110) sample upon exposure to H_2_ can be seen in Figure 6. Two new features appear at 143 and 157 meV upon dosing of 10 L & 100 L H_2_. A slight increase in energy is seen in both features at 100 L H_2_, to 145 and 163 meV. As previously seen with oxygen dosing, the residual C–O stretch mode persists at 261 meV. While this peak position decreases as with oxygen dosing, it redshifts to even lower energy, going from 261 to 249 meV. Unlike oxygen, the C–O modes do not completely disappear upon increased H_2_ dosing but are reduced in intensity by half. While researchers^58,59^ have found a 1 × 2 missing-row long-range reconstruction of the surface upon hydrogen adsorption on Pd(110) and Ni(110), it can be seen from our LEED images (Figure S3d–f in the Supporting Information) that we see no reconstruction, even at 100 L H_2_, which is expected on this quaternary surface.

The two hydrogen modes are similar in energy to those seen on other single-crystal surfaces. For example, on Co(101̅0)^60^ hydrogen modes appear at 135 and 145 meV, on Cr(111)^61^ at 112 and 155 meV, on Fe(110)^62^ at 131 meV, and on Ni(110)^63^ at 117 meV. Major differences can be seen in these hydrogen modes compared to the oxygen modes seen in Figure 4, primarily in the fwhm of these peaks. As mentioned earlier, M-O and M-O_*x*_ modes average around 35 meV fwhm. However, these two hydrogen modes have a fwhm of around 9 meV, closer to that of the elastic peak at 8 meV. This, together with the fact that only two distinct modes are seen, suggests that the elemental variation of adsorption sites does not lead to a significant difference in the vibrational energy of individual modes, as observed with oxygen. It should be noted that it is very difficult to measure H vibrational modes due to the weak dipole moment of adsorbed hydrogen.

### 3.6 Simulated Vibrational Spectra for H, O, and CO on HEA(110)

Previous theoretical work has identified *fcc* 3-fold hollow sites along the ridge as the most stable adsorption sites on the (110) facets of *fcc* metals for small atomic adsorbates. A local coverage of 1 ML of atomic O, for instance, has been shown to preferentially occupy the *fcc* sites in a zigzag configuration on Pt(110)^64^ and Rh(110).^65^ All *fcc* sites are occupied by atomic O at 2 ML, resulting in surface reconstruction.^65^ Similar findings have been reported for hydrogen adsorption on Ni(110),^63,66^ Pd(110),^58,67^ and Rh(110).^68^ On a compositionally disordered surface like the HEA(110), when an oversaturating amount of a strongly binding species such as atomic O is deposited on it, irregular reconstruction and possibly amorphization of the surface is expected to occur.

**Figure 7:**
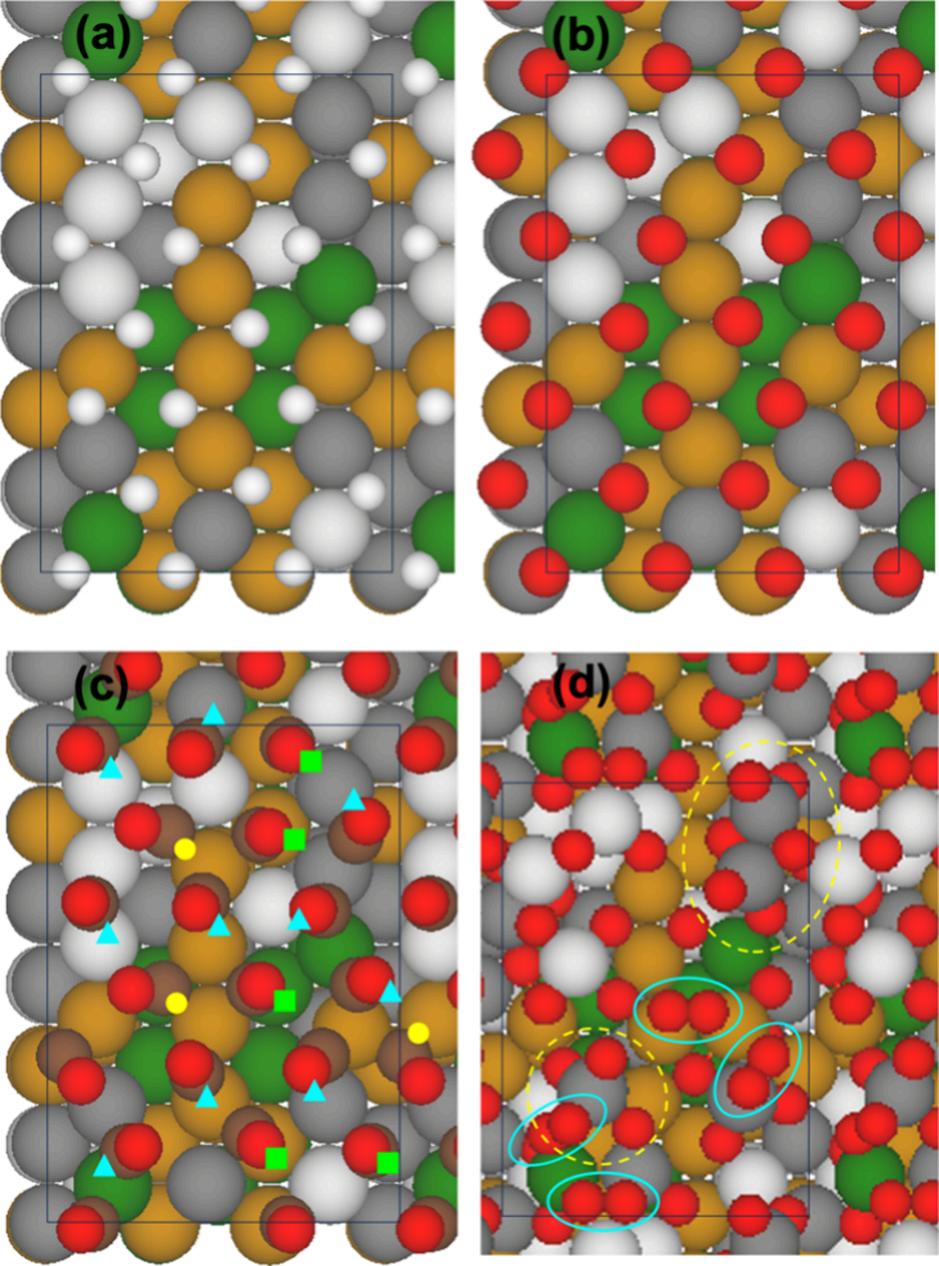
DFT-calculated minimum-energy configurations for a local coverage of 1 ML of (a) atomic H, (b) atomic O, and (c) CO; and (d) 2 ML of atomic O. The surface unit cell is outlined in each panel. Same color code as in Figure 1, with additionally C = brown. In panel (c), CO adsorbed on different sites are as indicated. In panel (d), O_2_ (solid blue lines) and CrO_*x*_ (dashed yellow lines) moieties are circled.

Without losing generality, we begin by simulating the vibrational spectra for 1 ML of atomic H, atomic O, and molecular CO on HEA(110). In the case of atomic O, we also present the results for 2 ML coverage. For each species, a given number of adatoms or ad-molecules are placed on the clean surface (Figure 1), and the surface is subjected to geometric optimization. The results are shown in Figure 7. At 1 ML the adsorption of neither atomic H nor atomic O causes notable changes to the surface (Figure 7a,b). Both species remain on *fcc* sites in the zigzag pattern. The adsorption of 1 ML CO, on the other hand, causes visible perturbation to the metal surface, and the molecules end up variously occupying on-top, bridge, and 3-fold sites (Figure 7c). The adsorption of 2 ML of atomic O significantly disrupts the top metal surface, resulting in the spontaneous formation of metal oxide (including CrO_*x*_, circled with yellow dashed lines in Figure 7d) and O_2_ (circled with blue solid lines in Figure 7d) moieties. The latter represents condensed oxygen at conditions represented by geometry optimization, i.e., absence of thermal energy. The corresponding adsorption energies are reported in Table 1, in comparison to HEA(111) and (100). The vibrational spectrum is then simulated for each converged minimum-energy structure.

**Table 1:** Calculated Average Adsorption Energy (Δ*E*, in eV) for 1 ML of H, O, and CO on Common Facets of the HEA^a^

	(110)	(111)	(100)
H	–2.82	–2.94^b^	–2.89^b^
O	–6.11	–5.32^b^	–5.36
O (2 ML)	–5.17		
CO	–1.69	–0.81	–1.00

a Δ*E* values are with respect to the same atom or molecule in the gas phase and averaged by the number of adsorbates.

b From ref (26).

**Figure 8:**
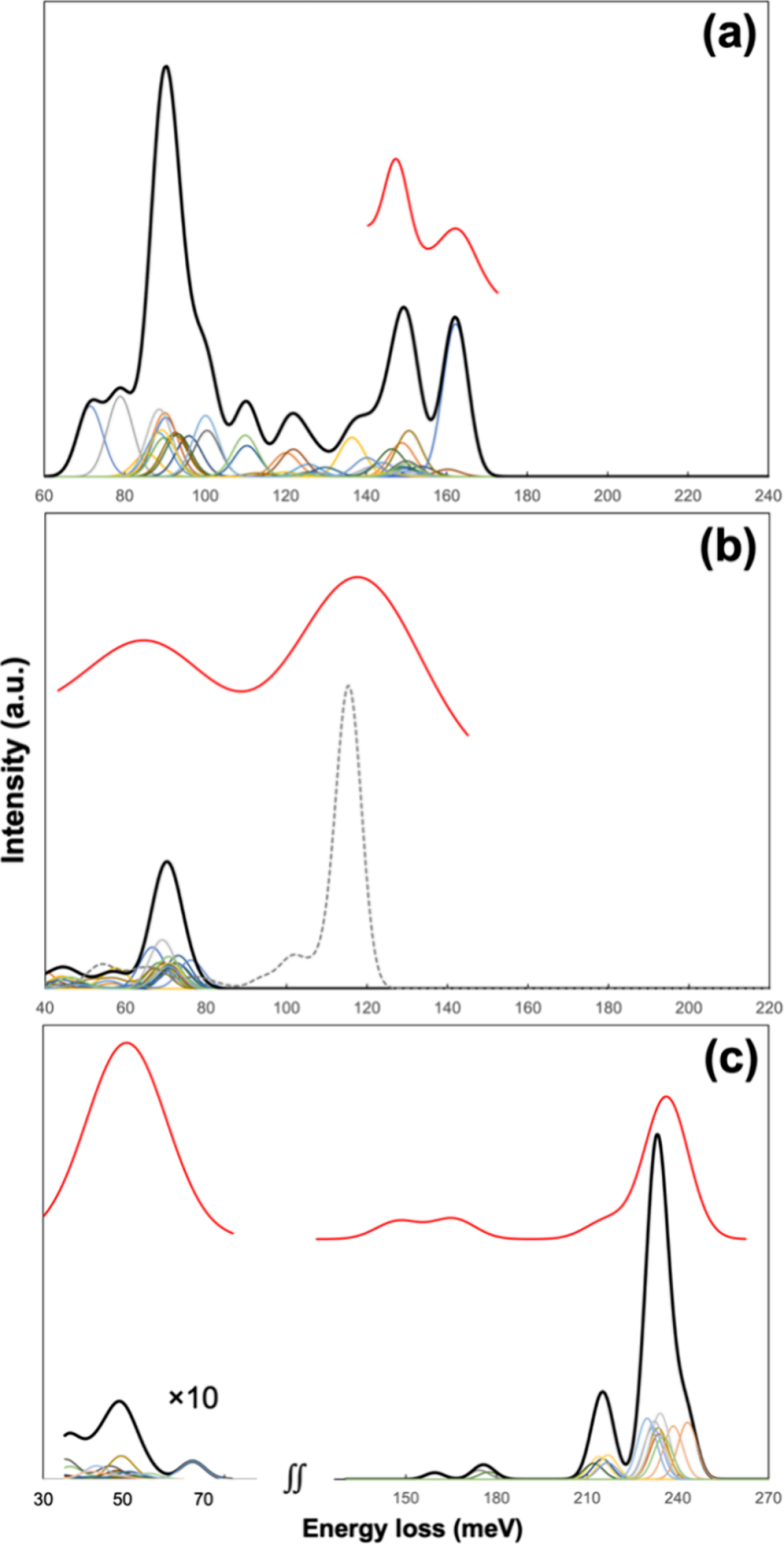
Simulated vibrational spectra for (a) 1 ML of H, (b) 1 and 2 ML of O, and (c) 1 ML of CO on the HEA(110) model. In each panel, the thin color lines are the contributions of individual atoms or molecules, and the thick black line represents their sum. The dashed line in panel (b) is 2 ML of O. The red curves are experimental data fits from Figure 6 (10 L H; with 5 meV blueshift), Figure 4 (50 L O_2_; 10 meV redshift), and Figure 3 (10 L CO with 30 meV redshift) for comparison.

The simulated vibrational spectrum for 1 ML of atomic H (Figure 7a) is shown in Figure 8a as the sum of the contributions of H atoms on all individual sites. The composite shows multiple peaks in the 70–160 meV region. While the heterogeneity in surface composition is expected to differentiate the vibrational frequencies of H atoms, the apparent structure in the spectrum is intriguing.

By analyzing the contributions from individual sites, we observe that more than one of the three fundamental modes of each H atom is dipole-active. This is due to the geometry of the (110) surface, in which the *fcc* sites are located on [111] microfacets that are oriented at an angle to the surface normal and due to the heterogeneity in surface composition. This is different from a flat, homogeneous surface such as the (111) facet of a monometallic *fcc* metal, where only the out-of-plane mode of adsorbed H atoms is dipole-active. Here, the out-of-plane mode has the highest frequency, but one of the two in-plane modes with a component aligned with the [001] direction is also active (here, plane refers to [111] microfacets). The mode that contributes the least to the spectral intensities is one of the in-plane modes that is aligned with the [11̅0] direction. The dipole-active modes are approximately clustered around 90 and 150 meV. As seen in Figure 8a, the appearance of the simulated spectrum between 140–180 meV bears a close resemblance to the HREELS spectra in Figure 6, wherein the fit to 10 L is overlaid and red-shifted by 5 meV to account for intrinsic DFT errors. The single high-intensity mode at 162 meV is due to H adsorbed on a Cr–Fe–Fe site, although H adsorbed on other Cr–Fe–Fe sites do not exhibit nearly as strong a feature at this energy. Overall, the double-peak structure at the leading edge of the spectrum is captured by our model and simulation.

The results for 1 ML of atomic O (Figure 7b) are similar in that O atoms on many of the *fcc* sites exhibit more than one active fundamental mode, including the out-of-plane mode that occurs at ca. 70 meV and is dominant, and one of the in-plane modes that has a [001] component in the 40–50 meV range with weaker intensities (Figure 8b). The spread in the active modes, 40–80 meV, is much narrower than with H, which coalesces into fewer separate features at the given resolution. Notably, no vibrational mode is seen beyond 80 meV. On the other hand, the simulated vibrational spectrum for 2 ML O (Figure 7d) exhibits a manifold of weak modes in 40–80 meV as before, and a new, more intense group of modes between 100–120 meV (Figure 8b, dashed line). It is qualitatively in close agreement with the HREELS spectra in Figure 4. A closer look at the different contributions reveals that the majority of the normal modes located at 40–80 meV with zero or small intensities are due to adsorbed atomic O, as in the spectrum for 1 ML of oxygen. The shoulder at ca. 100 meV is due to the O–O stretching of several O_2_ moieties, which are characterized as peroxide species based on the vibrational energy. The most intense contribution, which is located between 110–120 meV, originates from just a handful of modes due to CrO_*x*_ moieties. These theoretical findings support our interpretation that the vibrational features centered around 125 meV, which emerge at beyond 0.5 L of exposure to O_2_, are due to Cr oxide species.

The simulated spectrum for 1 ML of CO (Figure 8c) is qualitatively consistent with the HREELS spectra in Figure 3 with a 30-meV shift, and reveals the contributions of CO adsorbed on different types of surface sites. The spectrum is dominated by a strong feature that peaks at ca. 235 meV, together with a shoulder at ca. 215 meV and a couple of smaller features in 150–190 meV (Figure 8c). Another very small feature is located at ca. 50 meV. Analysis of the normal modes indicates that the 50 meV feature is molecular vibration against the surface, which has a wagging character due to the corrugated surface geometry. The rest of the modes, located at considerably higher energies, are all due to C–O stretching. The modes in 150–190 meV are due to CO adsorbed on 3-fold sites (labeled with yellow circles in Figure 7c). Those at ca. 215 meV are due to CO adsorbed at on-top sites in the trough (green squares, Figure 7c). The main contribution at ca. 235 meV is due to CO adsorbed on bridge sites on the ridge (blue triangles, Figure 7c). It is the most intense, both because of the higher intensities of the individual modes at this energy, and because more of the molecules are adsorbed on the bridge sites. The compositions of the sites are seen to have only a perturbative effect on the vibrational energy.

The blue- or redshift applied to the HREELS spectra for comparison to the calculated vibrational spectra (Figure 8) amounts to 3% and 8% of the energy of the highest-energy major vibrational mode for atomic H and O, respectively. Many theoretical studies^69−71^ have shown that, for gas-phase molecules, a scaling factor of 0.9 should be applied to DFT-calculated vibrational frequencies to obtain accurate predictions, i.e., deviations up to 10% are to be expected. In that light, the 30 meV shift, or 11% of the highest-energy mode, applied to the CO spectra in Figure 8 appears too large, particularly since DFT predicts the C–O stretching frequency on the individual component metals to within 2% of the experimental values.^44^ The relative positions (as well as intensities) of the C–O stretching modes along the energy axis for CO adsorbed on the different sites on the HEA(110) surface align closely with the experimental results, which confirms that CO adsorbs on multiple different sites as exist on an *fcc*(110) surface in the experiment. What accounts for the larger-than-expected deviation is, however, unclear. Increasing CO coverage is known to blueshift the C–O stretching frequency,^11,72,73^ but coverage alone cannot account for a 30-meV blueshift for CO adsorbed on a given site. Parameter convergence and effects of spin polarization were also checked, none of which diminished the deviation. Whether this discrepancy is indicative of further theoretical shortcomings at the GGA description of DFT remains to be seen. We are fully aware of the long-standing CO/Pt(111) puzzle,^74^ and that the consensus is converging toward it being a density-driven error involving incorrect charge transfer between the molecule and metal surface.^75^ A compositionally heterogeneous surface like the HEA possibly makes it even more difficult to describe the local density correctly. Nonetheless, even in the case of CO/Pt(111), while GGA incorrectly predicts the preferred adsorption site, the vibration frequency is nonetheless predicted quite accurately for each adsorption site.^44^

Despite the limited size of our model for the CoCrFeNi(110) surface, it closely captures the complex vibrational properties of H, O, and CO qualitatively on the HEA. In the cases of H and O, good quantitative agreement is also obtained. The theoretical results not only confirm the experimental assignments made above, but also help explain certain details of the HREELS spectra and provide insights into the local atomic structures that are responsible for the experimental observations. The agreement between our spectroscopic and theoretical results further indicates that the surface model provides a good representation of the chemical interactions of these common adsorbates with the HEA surface.

## 4 Conclusions

In this work, we compare experimental measurements of the vibrational modes of common gases (CO, O_2_, and H_2_) adsorbed on a high-symmetry, single-crystal quaternary HEA CoCrFeNi(110) surface to DFT calculations. Experimentally, this was achieved using HREELS following the adsorption of these gases. The symmetry of the sample surface was verified with LEED, which showed a large single-crystal grain oriented in the [110] direction. The DFT calculations were performed on a large (110) slab approximating this HEA surface, which consisted of a (6 × 6) surface unit cell and six metal layers. The slab was cut from a supercell representation of the bulk HEA constructed using the SCRAPs method.

Upon CO adsorption, C–O stretch modes typical of CO adsorption on bridge and on-top sites appeared. With higher dosing, they shifted to higher energies and additional modes appeared, which were attributed to CO bonded on 3-fold hollow sites. The width of these features indicates a large variety of inequivalent local electronic environments for each type of adsorption site, a result of the elemental heterogeneity of the surface. The simulated spectrum of 1 ML CO agrees with our experimental results, confirming that the adsorption of CO primarily occurs on bridge and on-top sites, with adsorption on 3-fold hollow sites contributing minor intensities.

HREELS experiments upon O_2_ dosing show two large modes which are attributed to the development of peroxide/metal oxide species. As with CO, the width of these features indicates that oxygen adsorbs on a variety of different sites that vary in their composition. Our LEED measurements show no additional superstructures. This is consistent with both HREELS measurements and DFT results. A comparison of HREELS measurements to the literature, our previous XPS measurements,^26^ and computational results suggests that the M-O_*x*_ modes initially consist of adsorption on sites that are rich in Cr. These theoretical findings support the interpretation that the vibrational features centered around 125 meV in our HREELS spectra are due to Cr oxide species.

Lastly, HREELS experiments upon H_2_ dosing reveal two modes around 140–160 meV, which we attribute to dissociative adsorption of hydrogen on 3-fold hollow sites. Unlike with the other adsorbates studied here, these features have a width similar to that of the elastic peak. DFT calculations agree, qualitatively, with our experimental results, showing two distinct modes between 120–200 meV. LEED reveals no ensuing H-induced reconstruction through saturation coverage.

In summary, our combined experimental and computational investigations have helped elucidate the adsorption properties of several common gases on this Czochralski synthesized, single-crystal, random quaternary alloy surface. This (110) alloy surface, which has structural symmetry but lacks compositional symmetry, presents significant compositional variation in different adsorption sites, which significantly affect the vibrational modes. While many other researchers have studied these adsorbates on many mono- and bimetallic surfaces, this study provides a unique view of gas adsorption on this quaternary random alloy surface.

## Supporting Information Available

Additional information on crystal growth and characterization. Additional LEED images at various O_2_ and H_2_ dosing (PDF)
