# Supplementary material for: Vibrational study of CO, O2, and H2 Adsorbed on the CoCrFeNi (110) High Entropy Alloy Surface
Source: J Phys Chem C Nanomater Interfaces. 2024 Aug 19;128(34):14315–25. doi: 10.1021/acs.jpcc.4c03938 (PMC11372746; doi:10.1021/acs.jpcc.4c03938)
Supplement: Supplementary file 1 — jp4c03938_si_001.pdf [file jp4c03938_si_001.pdf]

Vibrational Study of CO, O_2, and H_2 Adsorbed on CoCrFeNi (110) HEA Surface

Frank McKay[1], Andrew N. Okafor[2], David P. Young[1], Ye Xu[2], Phillip T. Sprunger[1*]

[1] *Department of Physics and Astronomy, Louisiana State University, Baton Rouge, LA 70803, USA*

[2] *Cain Department of Chemical Engineering, Louisiana State University, Baton Rouge, LA 70803, USA*

Supporting Information

*Corresponding author; email: phils@lsu.edu

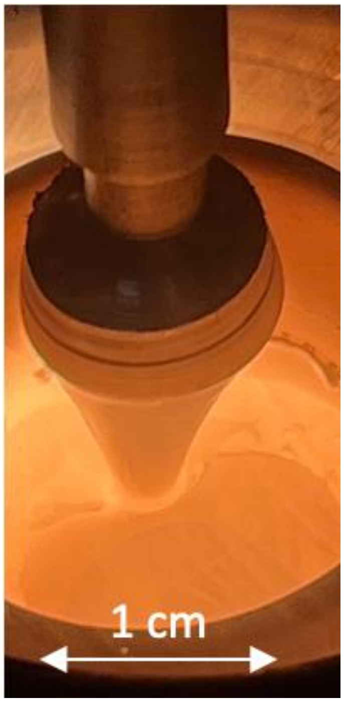

Fig S1: Modified Czochralski technique shown. CoCrFeNi single-crystal boule is being grown/pulled in an RF furnace. Seeding W single-crystal rod shown at top.

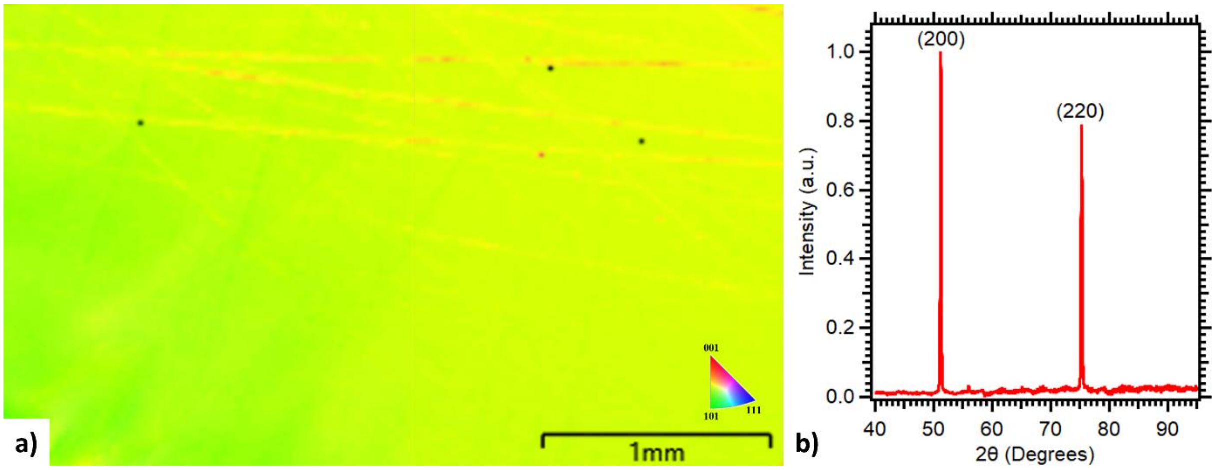

Figure S2: a) Two-dimensional EBSD composite image of CoCrFeNi (110) sample; the nearly monochromatic image of the large sample, stitched together from two large images, indicates a large, oriented grain. The horizontal lines are remnants of crystal polishing (dark spots are residual diamond polishing particles). b) XRD of CoCrFeNi (110) in sample-spinning mode.

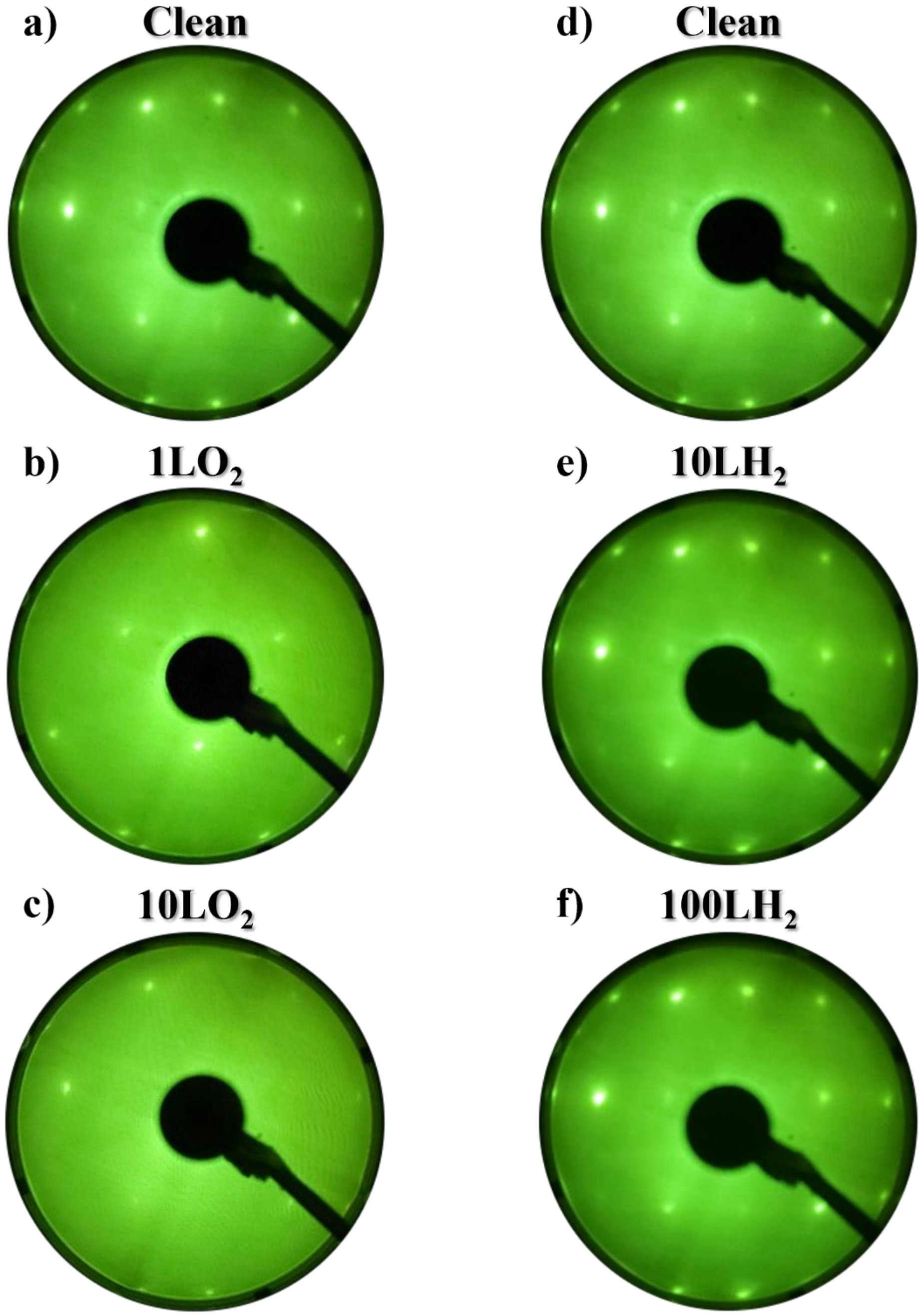

Figure S3: Low-energy electron diffraction (LEED) images of (at 221 eV) our CoCrFeNi (110) sample at LN2 temperatures. Upon oxygen dosing (a-c) any apparent symmetry is lost as the background becomes more diffuse. Upon hydrogen dosing (d-f) no additional symmetries appear or change in the symmetry seen before dosing.

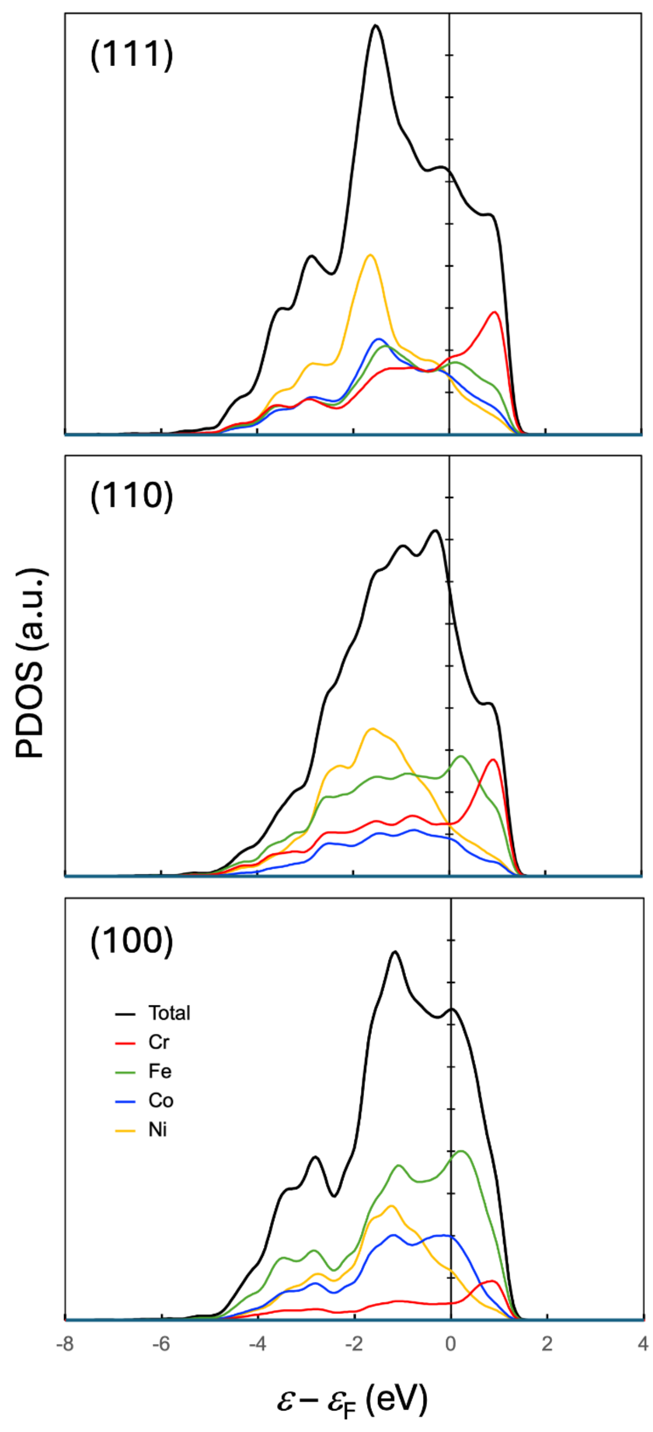

Figure S4: Projected density of states (PDOS) calculations of CoCrFeNi simulated surface for three facets: (111), (110), and (100).
